# Impact of a TB epidemic on worker and firm productivity: the regional perspective from Ukraine

**DOI:** 10.1007/s00148-026-01197-5

**Published:** 2026-09-14

**Authors:** Olena Nizalova, Oleksandr Shepotylo

**Affiliations:** 1 University of Kent, Canterbury, England; 2 Aston University, Birmingham, England

**Keywords:** Tuberculosis, Productivity, Regional wage, Total factor productivity, TB Epidemic, Sustainable development goals, O18, I15, J24

## Abstract

This paper investigates the indirect economic effects of tuberculosis (TB) in a high TB burden country, focusing on worker and firm productivity. Using unique administrative data from Ukraine, in 2003–2009, we find that TB prevalence leads to significantly lower wages and total factor productivity (TFP), while higher TB incidence, reflecting risk of exposure, is linked to higher wages and productivity. Additional analysis reveals strong spatial spillovers, underlining the contagious nature of the disease and the need for coordinated policy responses. Our estimates suggest that a 10% reduction in TB prevalence could yield a 1.05% GDP gain (0.15% from higher wages and 0.89% from higher TFP), compared to TB-related spending of only 0.04% of GDP that same year. These findings highlight the underinvestment in TB control and the broader economic stakes of epidemic containment.

## Introduction

Tuberculosis (TB) remains a major global health challenge, despite being both curable and preventable. According to the most recent Global TB Report (WHO 2024), TB has once again become the leading cause of death from a single infectious agent, having temporarily fallen behind COVID-19 during the pandemic. While TB primarily affects low- and middle-income countries, the globalisation of trade and migration ensures that it remains a threat to public health worldwide. In recognition of this, the United Nations convened a high-level meeting in 2018 titled “United to End TB: An Urgent Global Response to a Global Epidemic,” emphasising the need for coordinated international action. Sustainable Development Goal 3 aims to reduce TB deaths by 90% and incidence by 80% by 2030, while the End TB Strategy targets a 95% reduction in deaths and a 90% reduction in incidence by 2035 (WHO 2018, 2019). However, these targets are now unlikely to be met due to the setbacks caused by the COVID-19 pandemic (Cilloni et al. 2020; Kirby 2021). In 2023, 10.8 million people around the world had TB, up from 10.1 million in 2018, and 1.25 million people died from the disease (WHO 2024). Over 95% of the cases were registered in low- and middle-income countries.

Beyond its direct health consequences, TB is associated with escalating economic and social risks. Drug-resistant strains (multi-drug resistant (MDR) and extra drug-resistant (XDR)) are now reported in nearly every country. The disease is closely linked with HIV/AIDS, with an estimated one in four HIV-positive individuals dying from TB each year. Critically, TB often strikes during individuals’ most economically productive years, causing not only premature mortality but also extended absences from the workforce, increased caregiving burdens, and long-term earnings losses. In 2009 alone, nearly 10 million children were orphaned due to TB-related deaths (WHO 2009).

A range of intervention programs has been implemented globally to reduce TB incidence and prevent epidemics. These include direct measures such as directly observed treatment (DOT) and Bacille Calmette–Guérin (BCG) vaccination, as well as indirect strategies targeting poverty alleviation and health education. These interventions demand considerable public investment, making it essential to evaluate them in light of the full economic burden TB imposes, not only through medical costs, but also via its broader effects on productivity and economic development (Laxminarayan et al. 2007).

The full economic cost of TB comprises several components: (i) direct costs of diagnosis and treatment; (ii) GDP losses due to premature mortality; (iii) productivity losses among those directly affected (diagnosed or not) stemming from sick leave, absences, and reduced work capacity; (iv) indirect effects on productivity related to caregiving, stress of having sick family members, reduced incentives for education and hampered creativity and entrepreneurship; and (v) broader social adverse effects (e.g., orphaned children). Existing empirical evidence primarily addresses the first three components—treatment expenses, mortality-related output losses, and short-term earnings reductions during illness (KPMG 2017; Ahlburg 2000; Laxminarayan et al. 2007).

The last two dimensions remain largely unexplored, leading to underestimation of the full economic cost and potentially distorting cost-benefit analyses used to guide policy. For example, Lomborg (2018) ranks TB control among the top ten Sustainable Development Goals in terms of social and economic return per dollar spent, and Lomborg (2023) estimates a benefit–cost ratio of 46. Yet, these assessments account primarily for lives saved and omit other costs that may be significant, such as productivity losses among infected individuals. These costs may include reduced productivity among caregivers and bereaved family members, and negative impacts on firm-level performance through lower life expectancy, weaker incentives for education and entrepreneurship, and diminished innovation and creativity. If the scale of these indirect effects varies across Sustainable Development Goal priorities, placing TB control at sixth on the list (see Table 1 in Lomborg 2023) may lead to misinformed public investment decisions.

Despite tuberculosis being among the most devastating infectious diseases globally, it has received remarkably little attention in the economics literature. As documented by Bloom et al. (2022), TB ranks second only to HIV/AIDS in terms of global disease burden measured in disability-adjusted life years (DALYs). In fact, the mechanisms of the TB effects mirror those of HIV/AIDS, which have been far more thoroughly studied by economists focusing on labour supply effects and returns to human capital.

This paper addresses a significant gap in the literature by analyzing the impact of TB on worker and firm productivity, making four key contributions. This is the first study to investigate the indirect productivity effects of tuberculosis, which informs a better understanding of the full economic costs of the TB epidemic.

Second, we introduce a clear conceptual and empirical distinction between TB prevalence (a stock measure reflecting the number of individuals known to have the disease at the start of the year) and TB incidence (a flow measure capturing new diagnoses over the course of the year). We show that these measures represent different aspects of the disease burden and have distinct implications for productivity outcomes—a methodological advancement compared to previous studies, which relied solely on measures of TB incidence.

Third, our study leverages Ukraine’s distinctive empirical setting, which offers insights relevant to both developing and developed countries. Like many low- and middle-income countries, it has experienced a high TB burden and rising drug resistance since the 1990s. At the same time, Ukraine possesses institutional features more typical of high-income countries, including comprehensive case detection, mandatory X-ray screening, and high-quality administrative data linking health and economic outcomes. While TB prevalence is too low in most high-income countries to support identification, and data limitations constrain analysis in many low-income contexts, Ukraine combines sufficient disease burden with reliable data, enabling credible estimation of TB’s economic effects.

Finally, we explore spatial spillovers and inter-regional economic linkages, offering new insights into how health shocks can propagate beyond affected areas and generate broader economic costs associated with the TB epidemic.

We rely on a rich sub-national administrative data combining epidemiological data on TB-related indicators with socio-economic information from administrative statistics and business data for small administrative units (raions). Out of 669 total raions, the resulting analytical sample contains 609 raions over the period from 2003 to 2009.

The main findings are as follows. Ongoing TB epidemic imposes considerable indirect economic costs in the form of lost productivity and associated inefficiencies. Both firms and individuals in regions with higher TB prevalence exhibit significantly lower average wages and total factor productivity (TFP). Specifically, a 10% increase in the TB prevalence rate is associated with a 0.3% decline in wages and a 0.9% reduction in firm-level TFP. In contrast, consistent with the Compensating Wage Differentials theory (Rosen 1986), TB incidence, the risk of contracting the disease, is positively linked with both wages and TFP, after controlling for prevalence. This suggests a form of risk premium for individuals and firms operating in high-incidence environments. However, from the societal perspective, this premium can also be interpreted as a structural inefficiency arising from a negative externality of doing business in TB-affected regions (Jnawali et al. 2021), where firms incur higher labor and managerial talent acquisition costs that can limit their competitiveness and entry into more dynamic markets. Spatial analysis reveals strong geographic spillovers consistent with the infectious nature of TB and underscores the need for coordinated epidemic containment strategies. Overall, we estimate that a 10% reduction in TB prevalence would result in productivity gains equivalent to approximately 2.4 billion Ukrainian hryvnia (UAH), representing 0.15% of Ukraine’s GDP in 2014 in terms of wages, and 0.89% of GDP in terms of firm productivity. In that same year, the total TB-related expenditure (including the Global Fund contribution) was equivalent to only 0.04% GDP, suggesting a clear case of underinvestment. Exploring the spatial lags of TB prevalence and incidence, we find significant cross-regional spillovers, highlighting the contagious nature of the disease and the need for coordinated containment strategies beyond raions’ administrative boundaries.

The rest of the paper continues as follows. Section 2 looks into theory, empirical literature, and the Ukrainian context. Section 3 discusses the methodology, followed by the description of the data in Section 4. Section 5 presents and discusses the results. Section 6 concludes and gives policy recommendations.

## Background

### Theoretical considerations

#### TB and wages

The starting point to understand how tuberculosis (TB), or any other disease, affects worker productivity is Becker’s (1965) time allocation theory. When an individual contracts TB—a debilitating and often prolonged illness^1^—the time available for market work shrinks. Even if the person retains employment, absences due to illness lead to reduced earnings. Moreover, TB doesn’t only affect the infected individual. Household members, particularly women in traditional caregiving roles, often reallocate their time toward non-market caregiving. This substitution away from labor market participation (or reduced hours and effort at work) reflects not only a direct shift in time allocation but can also lead to lower wages as part-time workers usually earn lower hourly wages. These effects are further exacerbated via possible reduced investment in education and on-the-job training as explained by the various theories of human capital (Becker 1964; Schultz 1961; Mincer 1974).

Grossman’s (1972) model of health capital develops Becker’s 1964 ideas further and offers a new approach to understand how health impacts productivity. In this framework, health is both a consumption good (valued for its own sake) and a productive asset, enhancing individuals’ ability to work and earn. This model also introduces a new channel of the indirect impact of TB on caregivers. In the model, individuals invest in their health to increase productive time. Caregivers, according to this logic, may experience sleep deprivation, stress, and psychological strain, all of which degrade their own health stock. This in turn lowers the effort per unit of time worked—a dimension not always observable in hours or employment status, but often reflected in lower earnings, decreased on-the-job performance, and reduced upward mobility. Thus, both direct illness and caregiving responsibilities act as negative shocks to effective labor input, diminishing both labor supply (extensive and intensive margins) and human capital returns.

Strauss and Thomas (1998) extend this conceptualisation by emphasising the empirical link between health and productivity, particularly in low- and middle-income country (LMIC) contexts where health infrastructure is limited and informal care responsibilities are prevalent. Their work shows that health status influences not just participation in the labor force, but also productivity conditional on working. Applying this logic to TB, the presence of illness in the household can even depress wages through reduced effort intensity and higher job fragility. For instance, a worker who arrives late or is distracted due to caregiving duties may underperform, miss promotion opportunities, or experience employer discrimination, all of which can suppress wage trajectories. Moreover, Yew and Zhang (2025) explore theoretically the negative effects of health externalities on labour productivity, which play a significant role in the case of infectious diseases, such as TB, due to the risk of infecting others.

Additionally, Strauss and Thomas (1998) highlight the feedback loop: poor health leads to lower income, which in turn leads to fewer resources for health investment, resulting in sustained low productivity. This mechanism may be especially relevant for households dealing with a high-burden chronic condition like TB. In aggregate, this suggests that TB at the regional level may depress average wages not just through fewer workers, but through less effective work among those still employed.

Although the theoretical mechanisms outlined above apply broadly to long-term health conditions, any move from theory to empirical analysis requires careful consideration of measurement. The epidemiological literature identifies two measures, incidence and prevalence. The first one is the flow variable and the second is a stock variable. It seems logical that in the frameworks discussed above, what matters is the TB prevalence rate—the number of people living with a TB diagnosis at a particular point in time. However, in the specific context of tuberculosis (TB), most empirical studies to date have focused exclusively on incidence—a flow variable measuring the number of newly diagnosed cases over a period of time. Yet, it is clear that they cannot be used interchangeably. If all of the newly diagnosed TB cases were to be instantly cured, TB prevalence would have been low, caregiving would not be needed, and the effects on productivity would have been minimal.

Incidence reflects short-term shocks (the risk of being infected) while prevalence reflects a chronic burden. Moreover, biological mechanisms related to infectious diseases stipulate that in the case of TB, the prevalence rate and the incidence rate will be correlated: the number of infected people around will have an effect on the number of newly infected cases, and higher incidence will lead to higher prevalence. Hence, correlation alone would have been sufficient to motivate the inclusion of both measures into the model. However, because the incidence rate is a measure of risk, there is also an important theoretical difference in the nature of the two measures.

From a labor economics perspective, the presence of TB in a region may not only reduce productivity but may also affect wage structures through compensating differentials. Rosen’s (1986) theory of compensating wage differentials posits that workers require higher wages to accept jobs involving undesirable attributes, such as higher health risks or poor working conditions. Since TB incidence proxies for such latent health risks, employers may have to offer wage premiums to attract or retain workers in those settings.

However, the direction and magnitude of such compensating differentials depend on workers’ awareness of risk, employer’s bargaining power, and labor market flexibility. In informal labor markets or settings with weak labor protection laws, these compensating mechanisms may be muted or altogether absent. In such contexts, wages may not fully adjust to reflect health risk. Hence, it may be important to explore the heterogeneity of the effects across various dimensions.

In summary, TB prevalence and TB incidence influence regional wages through distinct and theoretically opposing mechanisms. The higher prevalence of tuberculosis reflects a sustained burden of the disease, which depresses wages through reduced labor supply, reduced work effort, and decreased investment in human capital, both for infected individuals and their caregivers. In contrast, a higher incidence of tuberculosis signals an increased risk of infection, which can lead employers to offer compensating wage differentials to attract or retain workers willing to operate in high-risk regions. Although prevalence captures the productivity-reducing effects of illness, incidence can be associated with wage premiums related to health-related job disamenities. Including both measures in the empirical analysis is therefore essential to disentangle these mechanisms and to avoid confounding productivity losses with risk compensation in wage and TFP dynamics.

#### TB and TFP

To analyze productivity at the firm level, we employ total factor productivity (TFP). It measures the efficiency with which an economy transforms inputs (labor and capital) into output. TFP is capturing factors like technological progress, organisational improvements, managerial talent, and human capital quality that are not directly accounted for by input quantities. It has been used extensively to study the impact of trade liberalisation, technological progress, and general firm performance (Bloom et al. 2012, 2013; Pavcnik 2002; De Loecker and Goldberg 2014). For our paper, TB prevalence, reflecting chronic disease burden, is expected to translate into lower TFP because of potentially lower quality of the workforce, poorer managerial talent, and strained organisational capacity. In contrast to wages, the theoretical effect of TB incidence on TFP is less straightforward. While one might conjecture a similar mechanism to compensating differentials, when firms respond to elevated health risks by adjusting work conditions or processes, the net effect on TFP remains unclear and requires empirical investigation.

### Relevant empirical literature

Evidence in the literature strongly points to the positive impact of population health on economic outcomes. However, the findings are difficult to compare because of the variation across several dimensions: level of the analysis (micro vs. macro), economic outcomes (GDP growth, income per capita, total factor productivity, individual wages), and health measures (population life expectancy, survival rates vs. specific disease indicators vs. individual health conditions). Moreover, the general discussion lacks the link with the specific disease—TB, which is subject to specific health interventions.

A recent review documents a wide body of evidence within micro-studies showing that better health—across a range of conditions from chronic disease to mental health—is consistently associated with higher earnings and stronger labour market participation across global contexts (Pinna Pintor et al. 2024).

Bloom et al. (2024) offer a review of the macro studies employing either life expectancy or survival rates, highlighting the differences in the effects when compared to the aggregated macro effects from micro studies. They argue that while micro-based studies (often based on wage regressions) focus on direct effects of individual health on earnings, macro-based models capture both direct and indirect effects, including those operating through education, capital accumulation, demographic transitions, and savings behavior. As a result, macro estimates are often substantially larger than micro-based ones with the exception of one strand of literature that finds no effect or even a small negative effect (e.g., Acemoglu and Johnson, 2007). Bloom et al. (2024) reconciles this discrepancy, sometimes called the “micro–macro puzzle,” carefully addressing conceptual and methodological differences, and finds that the estimates based on micro and macro studies are comparable.

To use a relatable example, a 1-year increase in the life expectancy of the population contributes to an increase in economic growth by approximately 4–7% (Barro 1996; Barro and Lee 1994; Barro and Sala-I-Martin 1995; Bloom and Canning 2000). However, those effects are heterogeneous across countries, and there is evidence of diminishing returns to health improvements (Bhargava et al. 2001).

A few studies in this literature investigate the impact of specific infectious diseases on economic growth. For example, estimates from a cross-country analysis over the period from 1965 to 1990 show that countries with intensive malaria grew 1.3% less per person per year and that a 10% reduction in malaria was associated with a 0.3% higher economic growth (Gallup and Sachs 2001). Brainerd and Siegler (2003) investigate the influence of the 1918 influenza pandemic on per capita income growth across different U.S. states and find a positive association during the 1920s, with one more death per capita associated with a 0.15% higher growth rate. At the same time, Karlsson et al. (2012) show that the 1918 influenza pandemic caused significant increases in poverty rates and a reduction in capital returns, with no discernible effects on earnings.

However, studies that specifically consider the effect of infectious diseases on economic growth are subject to one major criticism—potential endogeneity due to reverse causality, as rising incomes may be the cause of better prevention, treatment, and thus, higher life expectancy. To illustrate, a study by Datta and Reimer (2013) of the 100 endemic countries over the 17-year period shows that most of the earlier found effect of malaria is due to reverse causality, as rising incomes of the households allow for increased prevention and treatment of malaria. This concern is even more serious in the case of tuberculosis, as its onset is exceptionally closely related to poverty (Kamolratanakul et al. 1999; Peabody et al. 2005; Jackson et al. 2006). Moreover, upturns in TB cases and deaths are very likely in the periods of economic recessions (Nimalan and Dye 2010).

There are only two studies in the field of economics that focus on the effects of tuberculosis at the macro level. Delfino and Simmons (2005) apply the Lotka-Volterra type system capturing the dynamics of TB epidemics with a Solow-Swan growth model where output is produced from capital and healthy labour and find significant differences in the effect of an epidemic between rich and poor countries. According to the study, in high-income countries TB infection dies out quickly, while in less wealthy countries it converges to a steady state of positive prevalence of 2% of the population being infected. Moreover, in poor countries, there is evidence of a vicious circle with high TB rates generating a negative effect on economic growth and prosperity. Grimard and Harling (2004) address the issue of endogeneity in a cross-country setting by employing random- and fixed-effects models, as well as the correlated-effects generalised least squares (GLS) to model the impact of the average TB incidence on 5-year economic growth. Using a sample of 91 countries over the period from 1981 to 2000, they find that there is a persistent effect of 0.2 to 0.4 percent lower growth for every 10% higher incidence of TB.

The estimates of TB costs from micro-level studies in Thailand (Kamolratanakul et al. 1999) and the Philippines (Peabody et al. 2005), as well as for immigrant patients in the Netherlands (Kik et al. 2009), support this argument: infected people spend their savings, take loans from banks, borrow from relatives, and sell property in order to survive. However, among those micro-level studies, only (Peabody et al. 2005) was based on large-scale surveys.

Several studies have investigated the relationship between population health and total factor productivity (TFP), a key measure of economic efficiency. Cole and Neumayer (2006) use panel data from 52 countries and find that poor health (measured by malnutrition, malaria, and waterborne diseases) significantly reduces TFP growth. Similarly, Alvi and Ahmed (2014) estimate a Cobb-Douglas production function for 37 countries and show that life expectancy, used as a proxy for population health, has a strong and statistically significant positive effect on TFP, with larger effects in developing countries. The most recent study by Bloom et al. (2025) provides strong evidence for the negative effects of the COVID-19 pandemic on TFP. Although there is no study of the effect of tuberculosis on TFP, these findings provide support for our hypothesis that tuberculosis can have effects beyond individual productivity and hamper broader economic efficiency and firm-level performance.

To conclude, our paper contributes to the literature as follows. First, we estimate the indirect productivity costs of tuberculosis, using employment-weighted average TFP and wages at the region level to capture effects on both workers and firms. In contrast, earlier studies have focused either on accounting-based estimations of medical costs and household coping mechanisms (Kamolratanakul et al. 1999; Peabody et al. 2005), or on cross-country growth regressions using national aggregates (Delfino and Simmons 2005; Grimard and Harling 2004), without isolating productivity-specific mechanisms.

Second, with this study we make a methodological advancement by introducing a conceptual distinction between TB prevalence and incidence—respectively reflecting chronic burden and new infection risk—and demonstrate how these measures have distinct theoretical implications for productivity outcomes. Previous empirical work has typically relied only on TB incidence, which, given the relationship between the two measures, most likely resulted in biased estimates. This illustration of the importance of accounting for both measures that describe the disease burden can be instrumental for further reconciliation between the evidence on the effects of health on economic growth based on micro-level studies and that from macro-level ones.

Third, by exploiting sub-national variation within one upper-middle-income country with both a high TB burden and rich administrative data, this paper offers a rare opportunity to study the economic effects of an infectious disease using robust regional evidence. This setting allows us to overcome the challenges that prevent extensive research of the tuberculosis effects—poor data quality in low-income settings with a high TB burden and low disease prevalence in high-income countries that are rich in data, while avoiding the comparability issues inherent to cross-country studies.

Finally, given the infectious nature of the disease, we examine spatial spillovers and economic linkages across regions, providing new insights into how health shocks propagate beyond local boundaries and reveal higher costs of the TB epidemic.

### Ukrainian context

After the collapse of the USSR, Ukraine, as well as the other post-Soviet countries, has experienced continuous worsening of population health, including an enormous growth in tuberculosis prevalence (Vassal et al. 2009). This has been aggravated by a substantial under-financing of organisations responsible for tuberculosis control (Hammers and Downs 2003), increased poverty among the population, and the abolition of social security benefits for TB patients, such as disability pensions or job security (Drobniewski et al. 2004). Ukraine had a TB incidence rate of 127 per 100,000 population in 2004–2005, falling to 91 by 2015^2^ and to 84 by 2017 (WHO 2018). Nonetheless, Ukraine was among the 30 countries with the highest burden of multi-drug resistant TB (16% of newly registered cases being MDR TB compared to the average of 3.8% for this group of countries) (WHO 2015; Lytvynenko et al. 2014), with 30 cases per 100,000 population in 2017 (WHO 2018). The TB burden is affected by the spread of the HIV/AIDS epidemic (Ukraine contributes more than 20% of the newly diagnosed cases in Europe and Eurasia region (Vassal et al. 2009)), high rates of urbanisation (70% of Ukrainian population live in cities), immigration from countries with high rates of HIV and tuberculosis, growth of homeless and mobile population groups (Codecasa and Migliori 2004).

Two other concerns have adversely affected the situation in the region and call for heightened attention to TB control in Ukraine and neighbouring countries. One is the ongoing Russian aggression, which started in the summer of 2014 in three regions—Crimea, Donetsk, and Luhansk. Donetsk and Luhansk regions have historically been the most TB-burdened regions in the country. The ongoing military conflict implies, among other things, considerable disturbance to the TB surveillance and treatment due to the interruptions in the supply of medicine in the occupied territories. The latter are directly linked to the development of MDR and XDR forms of TB. This situation has significantly deteriorated since the beginning of the full-scale Russian invasion in February 2022, which had caused widespread displacement, destruction of healthcare infrastructure, disruption of TB diagnostic and treatment services, and reduced access to medications across multiple regions. Currently, there are 4.6 million internally displaced persons (IDP) in Ukraine,^3^ who are more likely to have been either infected by or in contact with those infected, stay in crowded shelters, be unemployed, live in poverty, and have worse access to health care. Moreover, Burman et al. (2018) highlight the challenge in retaining the health care workforce specialising in tuberculosis treatment, not only in the conflict zone but throughout the country. There was large external migration since the beginning of the war in 2022, leading to 6.2 million external migrants,^4^ creating shortages of labour, including among healthcare workers. The other concern are the consequences of the COVID-19 pandemic. Cilloni et al. (2020) considered the cases of Ukraine, India, and Kenya and estimated that a 2–3 months lockdown in early 2020 caused a “setback of at least 5 to 8 years in the fight against TB” due to delays with diagnostics and treatment arrangements (StopTBPartnership 2020). Since then, there had been several other lockdowns before the full-scale invasion in February 2024, which have probably overshadowed but also amplified the negative effects of the COVID-19 pandemic on TB.

Being based on pre-war and pre-pandemic data, the findings of this study are highly relevant. They provide a rare and rigorous quantification of the indirect productivity costs of tuberculosis in a high-burden upper-middle-income country, offering critical insight into the economic consequences of prolonged infectious disease exposure. These estimates can serve as a valuable benchmark for designing evidence-based health recovery strategies in post-war Ukraine and offer transferable lessons for other countries facing similar epidemiological or institutional challenges. Moreover, the methodological advancement developed here—combining epidemiological and economic data at a fine spatial level and distinguishing between disease incidence and prevalence—can inform future research and policy across diverse contexts.

## Methodology

### Modeling productivity

The impact of tuberculosis on the economy is derived from two measures of productivity—a regional total factor productivity (TFP), which is a labor-weighted average TFP of all firms in the region, and an average regional monthly wage. TFP is often seen as a driver of economic growth, while the average regional wage reflects the marginal product of labor when markets are competitive. The first has mostly been the subject of the analysis in the fields of industrial organisation and international trade, while the latter is analyzed in labor economics. Thus, our models rely on sources from both fields, subject to data availability. To address the issue of endogeneity, we will estimate the effect of TB measures and other control variables on outcomes, exploiting the panel structure of the data.

Therefore, the models of worker and firm productivity define respectively the average wage rate $ARW_{it}$ and total factor productivity $TFP_{it}$ for raion *i* at time *t* as a function of variables at time (t-1) describing TB situation $TB_{it-1}$, average quality of human capital and relevant socio-economic characteristic in the following way:

1 $$\begin{aligned}&\ln {ARW_{it}}={\alpha _{0}} + {\alpha _{1}}\ln {TB_{it-1}} + {\alpha _{2}}Educ_{it-1} + {\alpha _{3}}{Educ_{it-1}}^{2} \nonumber \\&\qquad \qquad \qquad + {\alpha _{4}}\ln {Death_{it-1}} + {\alpha _{5}}Unempl_{it-1} + {\alpha _{6}}Urban_{it-1} + {\alpha _{7}}Density_{it-1} \nonumber \\&\qquad \qquad \qquad \qquad \qquad \qquad \qquad \qquad \qquad \qquad \qquad \qquad \qquad \quad + {\beta _{8}}T_{t} + {\beta _{9}}Prison_{i} * T_{t} + {\alpha _{i}} + u_{it} \end{aligned}$$

2 $$\begin{aligned}&\ln {TFP_{it}}={\beta _{0}} + {\beta _{1}}\ln {TB_{it-1}} + {\beta _{2}}Educ_{it-1} + {\beta _{3}}{Educ_{it-1}}^{2} \nonumber \\&\qquad \qquad \qquad + {\beta _{4}}\ln {Death_{it-1}} + {\beta _{5}}Unempl_{it-1} + {\beta _{6}}Exp_{it-1} + {\beta _{7}}Imp_{it-1} + {\beta _{8}}Urban_{it-1} \nonumber \\&\qquad \qquad \qquad \qquad \qquad \qquad \qquad \quad \quad \qquad \qquad + {\beta _{7}}Density_{it-1} + {\beta _{8}}T_{t} + {\beta _{9}}Prison_{i} * T_{t} + {\alpha _{i}} + u_{it}, \end{aligned}$$

In this equation, the vector *TB* includes TB incidence rate per 100,000 people (newly diagnosed cases over the course of the corresponding year) and TB prevalence per 100,000 people (number of people living with a TB diagnosis at the beginning of that year) in a raion. *Educ* indicates the share of employees with higher education; *Death*—death rate in a raion as a control of overall population health; *Unempl*—unemployment rate; *Exp*—share of exports in total output; *Imp*—share of imports in total output; *Urban*—share of urban population; *Density*—population density; $T_{t}$—is the time trend and $Prison_{i}$ is an indicator signaling that there is a prison in raion *i*. $\alpha _{i}$ reflects the raion-level unobserved effects. It is important to include the dummy for the presence of a prison because the medical regional statistics do not include information from the penitentiary system. Yet, the TB incidence in prisons exceeded the same statistic for the civilian population by a factor of 30 (e.g., Cocco et al. (2022) report “1424.2 cases per 100,000 people, compared with 58.3 cases per 100,000 people in the general population”). Following the theoretical considerations as discussed above, the coefficient on TB prevalence is expected to be negative, while the coefficient on TB incidence is expected to be positive.

Estimating these models using ordinary least squares may lead to biased estimates of the coefficients of interest if unobserved, time-invariant characteristics of raions are correlated with both TB indicators and productivity outcomes. For instance, structural differences such as long-standing industrial composition (e.g., mining towns in the Eastern Ukraine) or geographic remoteness (e.g., in Carpathian Mountains or Crimea) could simultaneously influence TB prevalence and incidence (through exposure risk or health system capacity) and affect worker and firm productivity (through access to markets, skilled labor, and capital investment). If such factors are not adequately controlled for, the estimated effects of TB on productivity may capture these confounding influences. To address this concern, we adopt a fixed-effects estimation approach, which accounts for all time-invariant characteristics at the raion level.

### TFP estimation

Regional TFP is computed as follows. Consider a production technology of a single-product firm *j* at time *t* described by a production function

3 $$\begin{aligned} Y_{jt}=L_{jt}^{\alpha _{l}}K_{jt}^{\alpha _{k}}M_{jt}^{\alpha _{m}}\exp (\omega _{jt}+u_{jt}), \end{aligned}$$

where $Y_{jt}$ units of real output are produced using $L_{jt}$ units of labor, $K_{jt}$ units of capital, deflated by producer-price deflator, and $M_{jt}$ units of material inputs. $\omega _{jt}$ is firm-specific productivity, unobservable by an econometrician, but known to the firm before it chooses variable inputs. It includes, among other things, unobserved characteristics of the labor force, such as quality of human capital of the workforce, entrepreneurship, creativity, and managerial talent. We elaborate on this further in the paper. ${u}_{jt}$ is an idiosyncratic shock to production that also captures a measurement error. $Y_{jt}$ is not observable, because we do not know firm-specific prices, $p_{jt}$. Sales, $R_{jt}=p_{jt}Y_{jt}$, are known. To filter out demand shocks from the productivity measure, we introduce a constant elasticity of substitution demand system and estimate equation (3) by Olley and Pakes (1996), taking into account the relationship between output and price (Loecker 2011). Firm-level TFP is computed as

4 $$\begin{aligned} TFP_{jt}=(\ln {R_{jt}}-\beta _{L}\ln {L_{jt}}-\beta _{K}\ln {K_{jt}}-\beta _{M}\ln {M_{jt}}-\beta _{Y}\ln {Y_{gt}})\frac{\sigma _{s}}{\sigma _{s}+1} \end{aligned}$$

where $\beta _{f}=\frac{\sigma _{s}+1}{\sigma _{s}}\alpha _{f}$, for $f=\{l,k,m\}$, $\sigma _{s}$ is elasticity of substitution, and $Y_{gt}$ is total output of industry *g*, where firm *j* operates. Details on the TFP estimation can be found in Shepotylo and Vakhitov (2015). Furthermore, firm-level TFP estimates are aggregated to the level of the region as given by

5 $$\begin{aligned} TFP_{it}=\sum _{jt\in i}w_{jt}TFP_{jt} \end{aligned}$$

where $w_{jt}$ is the share of firm’s *j* employment in the total employment in the region *i*, and $TFP_{jt}$ is TFP of firm *j*. It may be argued that regional variation in TFP is driven by differences in economic structure across regions rather than due to firm- and individual-level differences. To address this issue, we also computed sector-specific TFP as given by

6 $$\begin{aligned} TFP_{k,it}=\sum _{jt\in i,k}w_{jt}TFP_{jt} \end{aligned}$$

where $$k=\{agriculture,industry, services\}$$.

### Spatial determinants of productivity and TB

In order to account for possible spillovers from TB into neighboring regions to worker and firm productivity, which may arise due to commuting and spatial spread of the disease, we augment the models by adding a spatial dimension. We account for cross-region effects by specifying a spatial weighting matrix W and adding spatial lags of our variable of interest as follows:

7 $$\begin{aligned}&\ln {ARW_{it}}=\alpha _{0} + \alpha _{1}\ln {TB_{it-1}} + \alpha ^{W}_{1}W*\ln {TB_{it-1}}+ \alpha _{2}Educ_{it-1} \nonumber \\&\qquad \qquad \qquad + \alpha _{3}Educ_{it-1}^{2} + \alpha _{4}\ln {Death_{it-1}} + \alpha _{5}Unempl_{it-1} + \alpha _{6}Urban_{it-1} \nonumber \\&\qquad \qquad \qquad \qquad \qquad \qquad \qquad \quad \quad \,\,+ \alpha _{7}Density_{it-1} + {\beta _{8}}T_{t} + {\beta _{9}}Prison_{i} * T_{t} + \alpha _{i-1} + u_{it} \end{aligned}$$

8 $$\begin{aligned}&\ln {TFP_{it}}=\beta _{0}+ \beta _{1}\ln {TB_{it-1}} + \beta ^{W}_{1}W*\ln {TB_{it-1}} + \beta _{2}Educ_{it-1} + \beta _{3}Educ_{it-1}^{2} \nonumber \\&\qquad \qquad + \beta _{4}\ln {Death_{it-1}} + \beta _{5}Unempl_{it-1} + \beta _{6}Exp_{it-1} + \beta _{7}Imp_{it-1} + \beta _{8}Urban_{it-1} \nonumber \\&\qquad \qquad \qquad \qquad \qquad \qquad \qquad \qquad \qquad \,\, + \beta _{7}Density_{it-1} + {\beta _{8}}T_{t} + {\beta _{9}}Prison_{i} * T_{t} + \alpha _{i} + u_{it} \end{aligned}$$

where *W* is a contiguity-based I×I spatial weighting matrix, and *I* is the number of regions. Its diagonal elements are equal to zero. An off-diagonal element, $w_{ij}$, is positive if and only if regions *i* and *j* share a common border. All neighbors are equally important, and the elements of the weighting matrix are row-normalised, so $\sum _{j=1}^{R}w_{ij}=1,\,\forall i=1...R$. As a result, for any TB-related variable *tb* in *TB*, a spatial lag of *TB*, expressed as W∗TB is interpreted as a simple average of *TB* in all neighboring regions. We assume that an element of the error term has the following structure: $u_{it}=u_{i}+\varepsilon _{it}$, where $u_{i}$ is a time-invariant regional effect, and $\varepsilon _{it}$ is an idiosyncratic shock in region *i* at time *t*. Equations (7) and (8) can be estimated by standard methods with regional fixed effects. The interpretation of the coefficients on spatial terms is straightforward. If TB measures in all neighboring regions increase by 1 percent, then ARW and TFP in region *i* increase by $\alpha ^{W}_{1}$ and $\beta ^{W}_{1}$ percent, respectively.

**Table 1:** Descriptive statistics

	2003	2004	2005	2006	2007	2008	2009	Total
Average regional wage, 2001 UAH	312.87	357.94	453.85	527.66	594.60	638.62	617.28	498.38
	(137.27)$^{1}$	(138.29)	(154.71)	(158.33)	(165.33)	(156.82)	(145.61)	(192.12)
Average regional TFP (weighted)	−0.32	−0.08	−0.03	0.06	0.10	0.17	0.25	0.02
	(0.31)	(0.29)	(0.27)	(0.26)	(0.26)	(0.23)	(0.25)	(0.32)
TB prevalence (per 100,000)	299.63	245.49	237.91	230.05	217.47	211.91	204.35	235.44
	(98.86)	(85.83)	(83.03)	(75.10)	(73.15)	(72.54)	(69.27)	(85.34)
TB incidence (per 100,000)	71.76	75.99	80.70	81.10	78.17	75.86	71.39	76.55
	(29.61)	(33.58)	(31.67)	(32.61)	(30.85)	(31.16)	(27.07)	(31.24)
Death rate (per 1000)	18.83	18.55	19.30	18.99	19.09	18.90	17.95	18.82
	(3.91)	(4.19)	(4.33)	(3.96)	(4.17)	(4.34)	(4.09)	(4.16)
Workers with higher education, %	17.34	18.4	19.66	20.71	23.57	25.22	27.09	21.62
	(5.43)	(5.81)	(5.97)	(5.96)	(7.69)	(8.21)	(8.72)	(7.66)
Unemployment rate, %	5.47	5.65	5.25	4.61	4.03	5.30	3.11	4.79
	(3.24)	(3.28)	(3.05)	(2.79)	(2.45)	(2.63)	(1.60)	(2.91)
Urban population, %	44.18	43.73	44.1	44.37	45.52	42.17	42.07	43.79
	(31.04)	(31.00)	(31.02)	(30.87)	(31.49)	(29.34)	(29.23)	(30.61)
Population density	441.67	439.93	441.84	442.99	442.84	402.9	393.9	430.33
	(1049.14)	(1048.84)	(1049.34)	(1051.48)	(1007.71)	(990.72)	(979.16)	(1025.86)
Prison	0.22	0.21	0.21	0.22	0.21	0.19	0.20	0.21
	(0.41)	(0.41)	(0.41)	(0.41)	(0.41)	(0.40)	(0.40)	(0.41)
N obs	493	530	529	519	541	472	471	3555

Mean values are shown, with standard deviations in parentheses. The sample covers 609 raions observed annually from 2003 to 2009 (*N* = 3555 raion-years). Average regional wages are stated in 2001 constant Ukrainian hryvnia (UAH), deflated with the national CPI, while total factor productivity (TFP) is the labour-weighted average of firm-level estimates. Tuberculosis (TB) prevalence and incidence are reported as cases per 100,000 population. Socio-economic data are from the State Statistics Service, and TB indicators come from the Ministry of Health surveillance system

This model may suffer from misspecification due to the presence of other spatial effects, which, if omitted, are subsumed as part of the error term and correlate with the included spatial variables. In order to account for such effects, we introduce a spatial autocorrelation model (Anselin 1988). It adds a spatial lag of the dependent variable, *Wy*, as one of the controls:

9 $$\begin{aligned}&\ln {ARW_{it}}=\alpha _{0} + \alpha _{1}\ln {TB_{it-1}} + \alpha ^{W}_{1}W*\ln {TB_{it-1}}+ \alpha _{2}Educ_{it-1} + \alpha _{3}Educ_{it-1}^{2} \nonumber \\&\qquad \qquad \quad \quad + \alpha _{4}\ln {Death_{it-1}} + \alpha _{5}Unempl_{it-1} + \alpha _{6}Urban_{it-1} + \alpha _{7}Density_{it-1} \nonumber \\&\qquad \qquad \qquad \qquad \qquad \qquad \qquad \qquad +\rho *W*\ln {ARW_{it}} + {\beta _{8}}T_{t} + {\beta _{9}}Prison_{i} * T_{t} + \alpha _{i} + u_{it} \end{aligned}$$

10 $$\begin{aligned}&\ln {TFP_{it}}=\beta _{0}+ \beta _{1}\ln {TB_{it-1}} + \beta ^{W}_{1}W*\ln {TB_{it-1}} + \beta _{2}Educ_{it} + \beta _{3}Educ_{it-1}^{2} \nonumber \\&\quad \quad \quad \quad + \beta _{4}\ln {Death_{it-1}} + \beta _{5}Unempl_{it-1} + \beta _{6}Exp_{it-1} + \beta _{7}Imp_{it-1} + \beta _{8}Urban_{it-1} \nonumber \\&\qquad \qquad \qquad \quad + \beta _{7}Density_{it-1} + {\beta _{8}}T_{t} + {\beta _{9}}Prison_{i} * T_{t} + \lambda *W* \ln {TFP_{it}} + \alpha _{i} + u_{it} \end{aligned}$$

where ρ<1 and λ<1 are estimated spatial lag coefficients. The spatial lag variable *Wy* is an endogenous variable. In order to estimate this model, we use the instrumental variable approach, where the spatial lag of the dependent variable is instrumented by the spatial lags of all right-hand side exogenous variables (Kelejian and Prucha 1998).

**Fig. 1:**
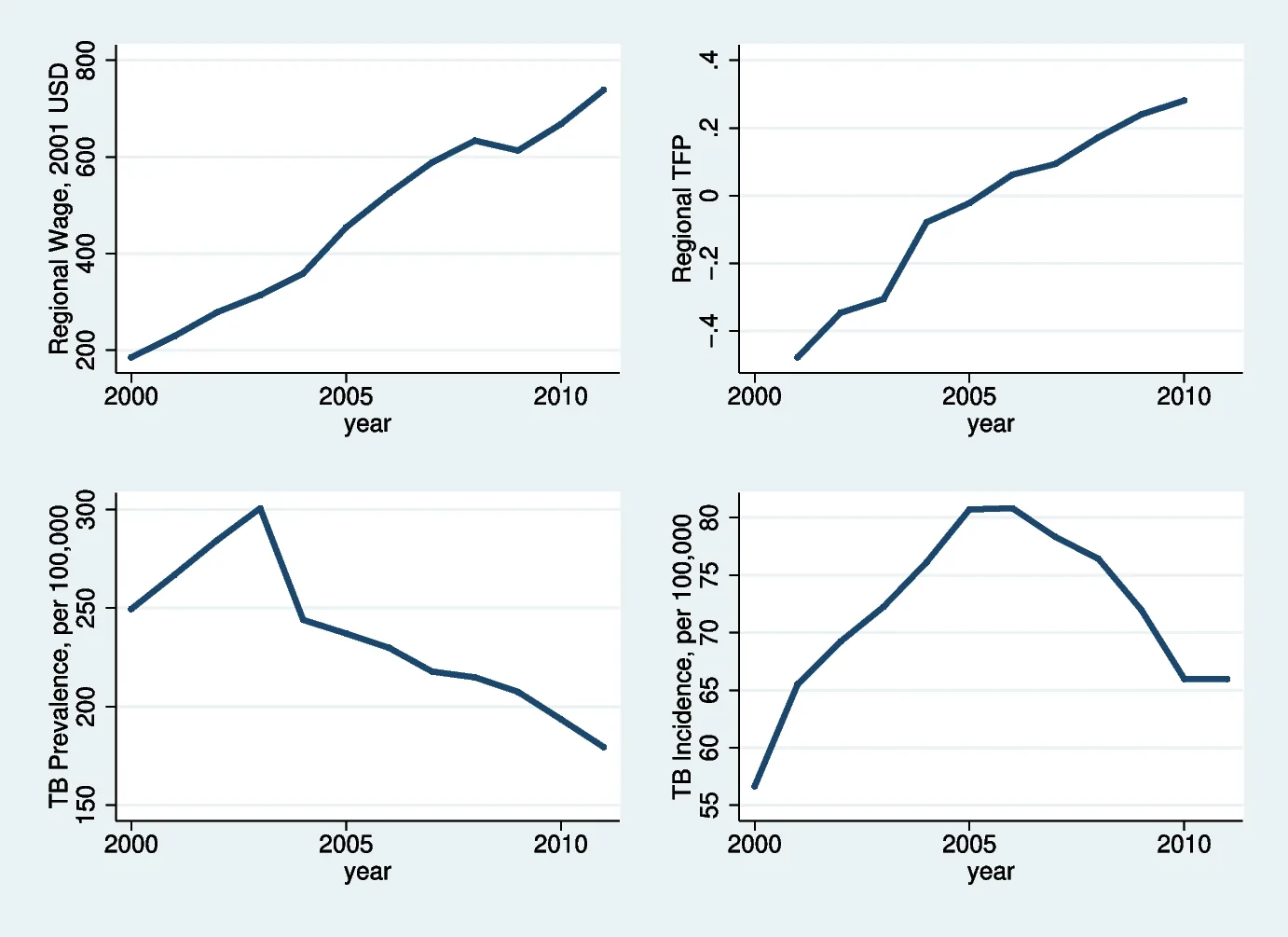
Time dynamics: measures of productivity and TB

**Fig. 2:**
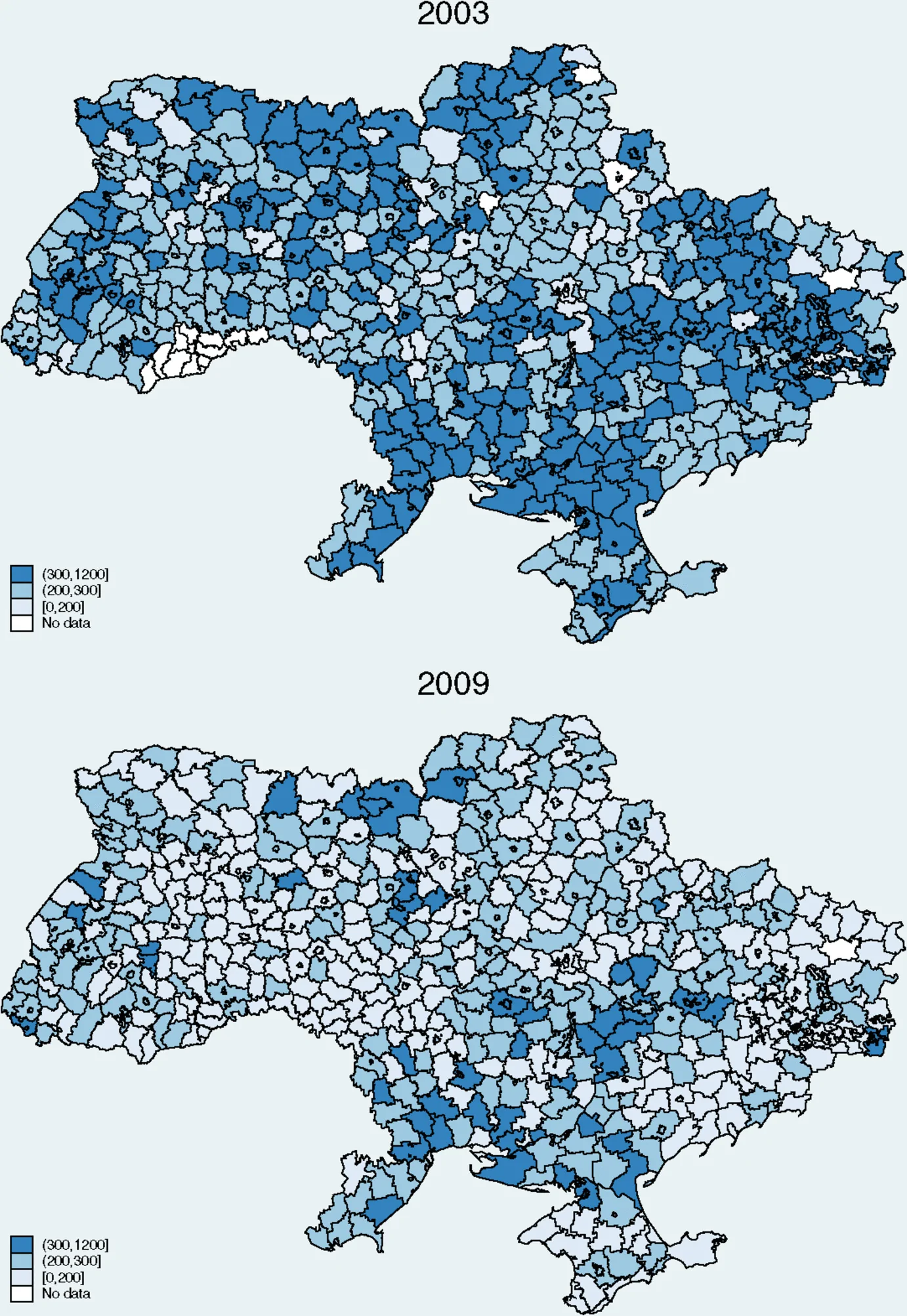
Regional variation in TB prevalence, 2003 vs. 2009

**Fig. 3:**
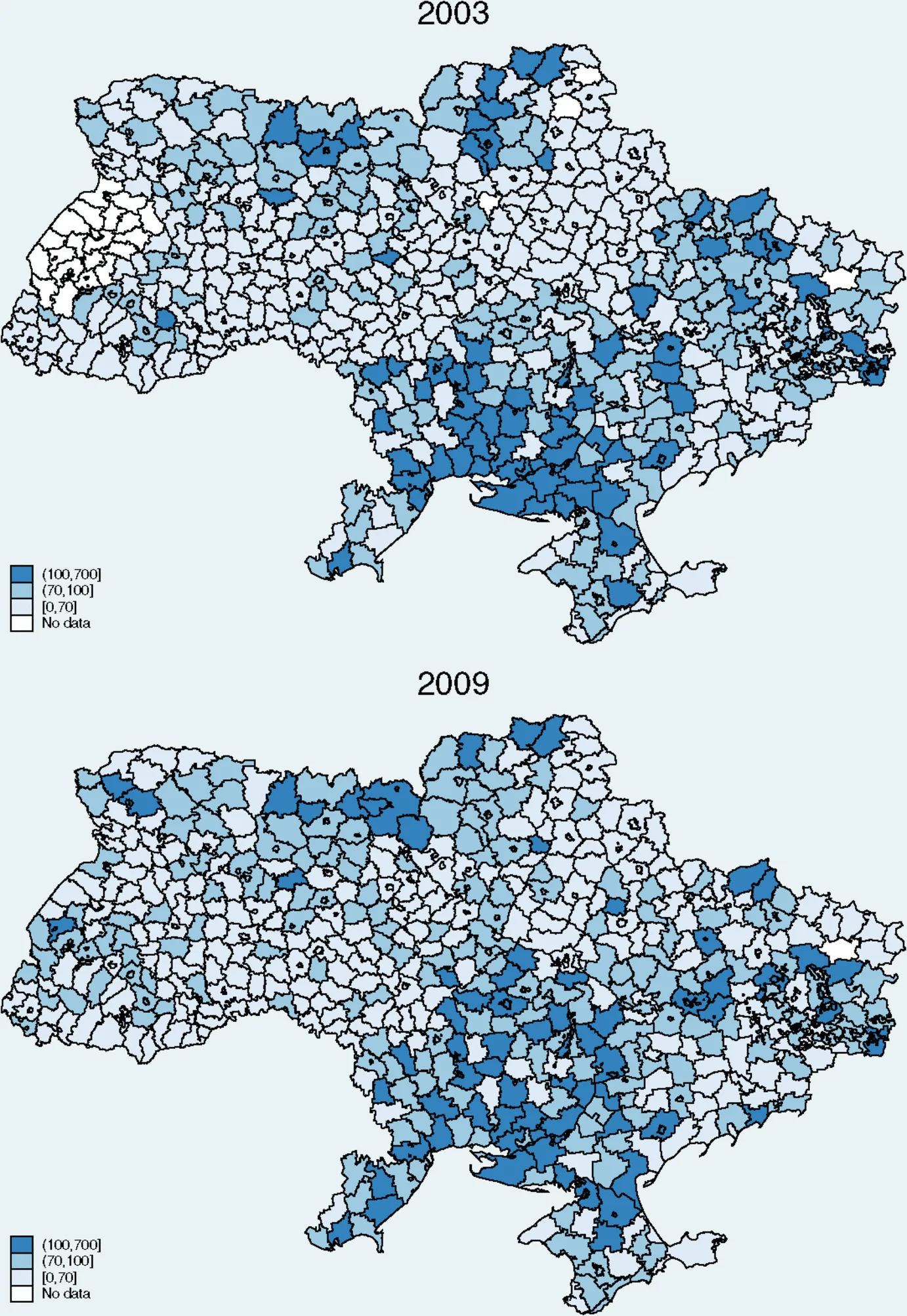
Regional variation in TB incidence, 2003 vs. 2009

**Fig. 4:**
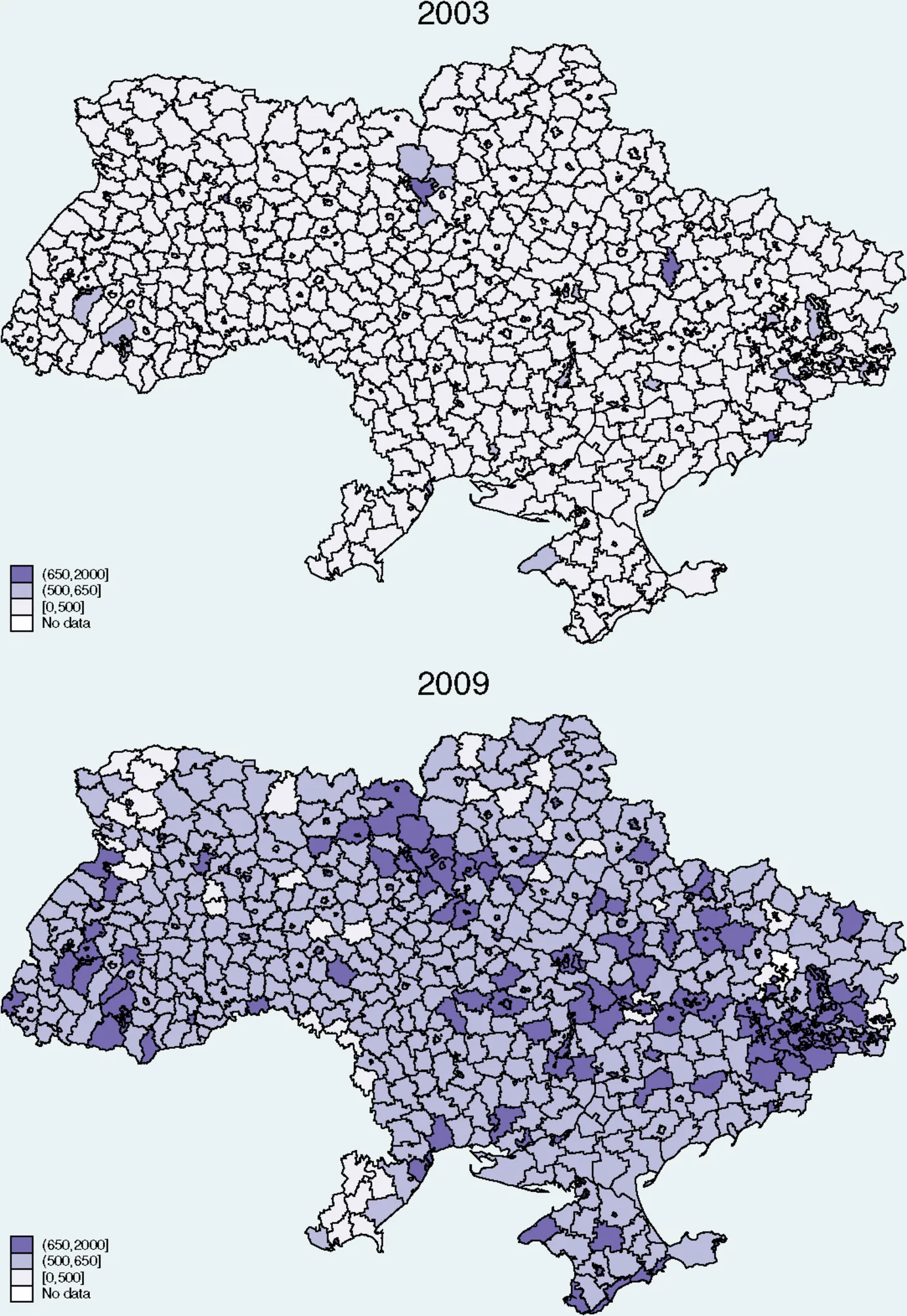
Regional variation in average wage, 2003 vs. 2009

**Fig. 5:**
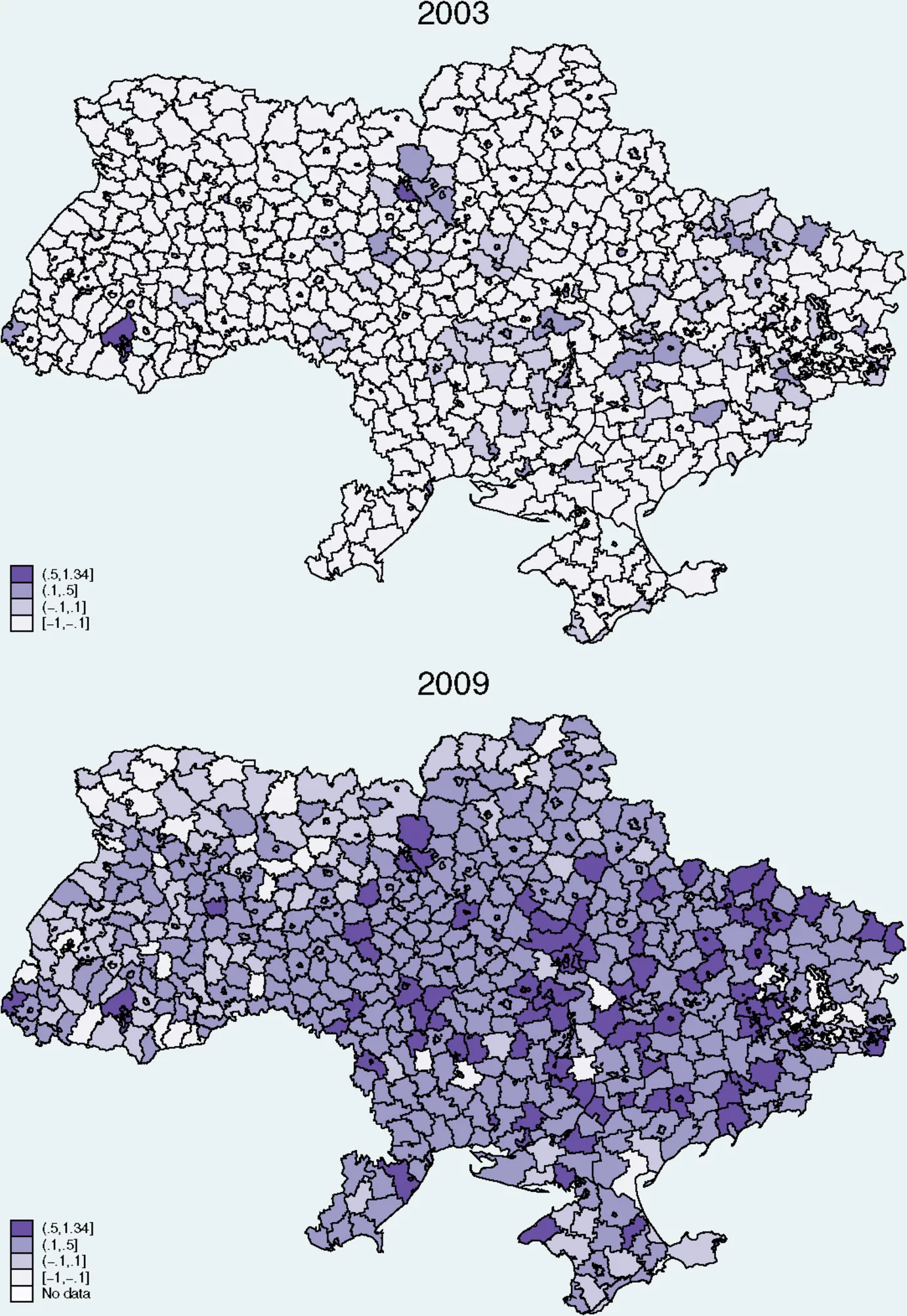
Regional variation in residual TFP, 2003 vs. 2009

## Data

We are using raion-level data collected by the Ukrainian Oblast Centers of Statistics for the period from 2003 to 2009. Table 1 provides information on the average sample characteristics across time, with each column providing information on current productivity and TB measures and other control variables. As can be seen, both the average regional wage (in 2001 constant prices) and the total factor productivity increased over the considered period. TB prevalence per 100,000 population decreased, while the incidence rate peaked in 2006 and then fell by the end of the period.

Total factor productivity for a region is the labor-weighted average TFP across all firms in the region. Firm-level TFP are recovered from the production functions estimated separately for each industry (2-digit NACE classification) using the Olley-Pakes procedure (Olley and Pakes 1996) controlling for sub-industry-specific demand and price shocks (Loecker 2011). The data for the study come from several statistical statements annually submitted to the Ukrainian Statistics Service by all firms in agricultural (NACE 01–03), industrial (NACE 04–33), and service sectors (NACE 34–96). For a more detailed description of the data, see Shepotylo and Vakhitov (2015). The data on average regional wage is routinely collected by the State Statistics Service from all the enterprises of all forms of ownership, including the agricultural sector. Self-employed people are not taken into account by either measure.

Figure 1 presents the dynamics of the key indicators, and Figs. 2, 3, 4, and 5—maps for TB measures and outcome variables at the start and the end of the sample period. As can be seen, over the considered period both average regional wages and average regional TFP have been steadily increasing, while both measures of the TB epidemic have seen a significant increase followed by a decline. Figures 2, 3, 4, and 5 portray three facts. One is that many regions in Ukraine have observed a considerable decline in the TB prevalence rates from 2003 to 2009, with a few remaining pockets of the TB prevalence exceeding 300 per 100,000 in the Northern and South Eastern parts of the country. At the same time, the regional pattern of the TB incidence has shown little change, with the Northern and South Eastern regions showing the highest concentrations of raions with the TB incidence exceeding 100 per 100,000 population. The dynamics of the regional distribution of both worker and firm productivity represent puzzling evidence, with regions with pockets of the highest productivity observed in the regions with the highest TB prevalence and incidence—in the North and South East of the country. However, this evidence is descriptive and requires a more elaborate analysis that follows.

## Estimation results

### Impact of TB on workers’ productivity

Table 2 presents the results for both ordinary least squares and fixed-effect estimation models. The first two columns show the effect on the average regional wage. The OLS model shows no significant effect of TB prevalence on the average regional wage. And, as the FE model shows, this turns out to be an underestimate of the true negative effect—a 10% higher TB prevalence decreases the ARW by 0.3%. Similarly, the OLS estimate of the effect of TB incidence (0.04) is lower than that from the FE estimation (0.05)—a 10% higher TB incidence leads to a 0.5% higher ARW. The effect of the prison presence is negative and increasing over the sample period. It is difficult to interpret this effect as the detailed epidemiological data on the prison population is not available.

**Table 2:** Estimated effect of TB incidence and TB prevalence

	Log regional wage		Residual log regional TFP		Residual log regional TFP - FE		
	OLS	FE	OLS	FE	Agr	Man	Serv
	(1)	(2)	(3)	(4)	(5)	(6)	(7)
Log of TB incidence	0.0438**	0.0548**	0.0423**	0.0506***	0.0340	−0.0021	0.1338
	(0.0171)	(0.0095)	(0.0185)	(0.0148)	−0.0755	(0.1298)	−0.3590
Log of TB prevalence	−0.0098	−0.0333**	−0.0467+	−0.0893***	0.0991	−0.0122	−0.0811
	(0.0260)	(0.0148)	(0.0272)	(0.0187)	−0.1273	(0.1760)	−0.2945
Log of death rate	−0.1212**	0.6259***	−0.0675	0.0846	−0.9345***	0.5343	1.7950*
	(0.0512)	(0.0490)	(0.0502)	(0.0695)	(0.3020)	(0.6631)	(0.9206)
% Emp with HE	0.0073***	0.0071**	0.0018	−0.0017	0.0110	−0.0139	−0.0638
	(0.0024)	(0.0034)	(0.0027)	(0.0029)	(0.0131)	(0.0298)	(0.0494)
% Emp with HE, squared	−0.0001***	−0.0001***	−0.0000	0.0001	−0.0001	−0.0000	0.0008*
	(0.0000)	(0.0000)	(0.0000)	(0.0000)	(0.0001)	(0.0005)	(0.0005)
Unemployment	−0.0191***	0.0074***	−0.0051	0.0006	0.0001	0.0151	−0.0933
	(0.0027)	(0.0017)	(0.0033)	(0.0026)	(0.0124)	(0.0242)	(0.0640)
Urban population	0.0040***	0.0008	−0.0013**	0.0009	−0.0057	−0.0015	0.5173**
	(0.0004)	(0.0008)	(0.0006)	(0.0015)	(0.0046)	(0.0043)	(0.2072)
Population density	0.0000	0.0001	0.0000	0.0003	0.0021**	0.0001	−0.0008***
	(0.0000)	(0.0001)	(0.0000)	(0.0003)	(0.0009)	(0.0004)	(0.0002)
Export share			−0.0221	−0.0130	−0.1677	0.4197	−0.5503
			(0.0593)	(0.0629)	(0.2200)	(0.3021)	(0.8000)
Import share			0.0083	−0.0404	−0.1239	0.2805**	0.5834
			(0.0504)	(0.0363)	(0.1049)	(0.1385)	(0.6492)
Prison × year	0.0008	−0.0181***	0.0062+	−0.0194***	−0.0665**	0.0445	0.1141
	(0.0033)	(0.0036)	(0.0037)	(0.0046)	(0.0285)	(0.0385)	(0.0699)
Year	0.1235***	0.1408***	0.0771***	0.0810***	0.3137***	0.1621***	0.0111
	(0.0036)	(0.0039)	(0.0042)	(0.0032)	(0.0179)	(0.0253)	(0.0736)
Constant	−241.6447***	−278.4420***	−154.2850***	−162.4863***	−629.3732***	−328.3743***	−58.5966
	(7.3674)	(7.9130)	(8.3469)	(6.4620)	(36.0297)	(51.0530)	(141.9271)
Number of observations	3555	3555	3555	3555	2340	1745	817
R-squared/F-stat	0.6922	941.49	0.2825	179.62	62.72	8.14	6.13
Number of id		609		609	562	504	311

Notes: Dependent variables are log average regional wage and residual log regional TFP growth; estimated with OLS (Columns 1 and 3), raion fixed effects (Columns 2 and 4), and sector-specific FE models for agriculture, manufacturing, and services (Columns 5–7). All right-hand side variables in all regressions are lagged 1 year. Robust standard errors in parentheses *p < 0.1; **p < 0.05; ***p < 0.01

Our set of control variables serves to isolate the TB-specific channels from broader regional correlates. Mortality rates absorb general health conditions that might otherwise confound the TB–productivity relationship. Unemployment controls for labour market slack that could independently affect wages. Urbanisation and population density capture agglomeration effects and differences in economic structure. By conditioning on these factors, the TB coefficients capture disease-specific productivity effects rather than general regional development patterns. The effects of the control variables on the outcomes are as expected. The share of employees with higher education has a positive but diminishing effect on wages. Mortality as a measure of the overall population health is positively associated with the average wage in the model when we control for raion fixed effects, which can point to the risks and corresponding compensating wage differentials. The unemployment rate is negatively associated with the average wage, which is consistent with the Efficiency Wage model (Akerlof and Yellen 1987).

### Impact of TB on firms’ productivity

We further explore the firms’ productivity and find that TB prevalence dampens firms’ productivity in the same manner as workers’ productivity. A 10% higher TB prevalence is associated with 0.9% lower TFP in the region. At the same time, a higher TB incidence rate has a positive and significant effect when we control for raion fixed effects. In this case, the same 10% increase in the TB incidence rate is associated with 0.5% higher total factor productivity.

To explore potential differences in the effect of TB epidemic on productivity across different sectors, we explored separately TFP in agricultural, manufacturing, and service industries. However, as the results are not statistically significant in these specifications (most likely due to the significantly reduced sample sizes), they will not be used in further analysis.

With regard to the control variables, all of the local population characteristics have no statistically significant impact on the firm-level productivity, which is reasonable, given that TFP is the residual productivity after controlling for labour, capital, and raw materials. Only the presence of prisons has been representing a dampening effect on productivity as measured by TFP, growing in magnitude with time.

### Spatial effects

We next model the spatial dependencies, as the spatial correlation in observed and unobserved variables may lead to the biased estimation of the effect of the TB variables on outcomes. As discussed in the literature (Kelejian and Piras 2017), this allows us to disentangle the effects reported in the previous section into the local and regional effects of the TB shocks. A better understanding of the mechanisms of the effect helps to design more targeted policy responses. High spatial spillovers would suggest wide regional policy responses, while stronger localised effects would indicate the need for very geographically targeted policies.

Since TB, like any other contagious disease, rapidly spreads across geographical boundaries, it is natural to include in the model the spatial lags of the TB prevalence and incidence. It will allow us to disentangle the effects of TB on wages and productivity into the local impact, stemming from the TB situation in raion *i*, and the spillovers, which are due to the TB situation in the neighbouring raions, adjacent to *i*. It is particularly important for policymakers to determine the optimal scale of policy interventions. A stronger local impact would deem relatively more important local, raion-level interventions, while a stronger neighbouring effect requires oblast and nationwide approaches. This approach leads to the spatially lagged X model (SLX) specification:

11 $$\begin{aligned} y=X\beta +WX\theta +\epsilon \end{aligned}$$

where *y* is an *N* vector of the dependent variables, *N* is the number of the spatial units, *X* is N×k matrix of the model variables, including the constant term, *W* is N×N spatial weighting matrix that represents the spatial links between the spatial units, which is row-normalised, $\sum _{j=1}^{N} w_{ij}=1$ for any i=1,..,N. The row-normalisation is important for the correct interpretation of the results, such as the spatial stability (stationarity) and economic size of the impact. ϵ is an *N* vector of error terms, β and θ are *k*-dimensional vectors of the coefficients and spatially-lagged coefficients.

Second, due to labour mobility, it may be important to account for the interactions of the labor markets in raions, as wages and productivity are endogenously determined as firms and workers interact across raions as well as higher-order spatial spillovers. This leads to a spatial autoregressive model (SAR) specification:

12 $$\begin{aligned} y=\rho Wy +X\beta +\epsilon , \end{aligned}$$

where |ρ|<1 is the spatial lag of the dependent variable, which captures spatial interactions. The condition that the spatial lag is less than 1 in absolute value is crucial for the model stability and interpretation of the results, as the model is not spatially stationary if the condition does not hold. It can also be shown that when the model is spatially stable, the SAR model can be represented as

13 $$\begin{aligned} y=\sum _{k=0}^{\infty } \rho ^k W^{k} X\beta + \sum _{k=0}^{\infty } \rho ^k W^{k} \epsilon \end{aligned}$$

which shows how the SAR model is related to the SLX model, as it captures not only the first-order spatial interactions but also the second and higher-order spatial effects, as well as the spatially correlated error terms. It becomes clear by comparing equations (12) and (13) that SLX model is an approximation of the SAR model when a) the higher order spatial effects, such as the TB situation in the raions, which are adjacent to raions that are adjacent to the raion *i* (second order spatial effects), are weak and b) there is no/weak spatial correlation in the error term.

Table 3 presents results from the fixed effects estimation with the spatial lags of TB with (SAR model) and without (SLX) controls for the spatial lag of the dependent variable. Columns (1) and (3) show that, in general, TB in the neighbouring regions has a similar qualitative effect on both wages and firm productivity as TB in the region itself—a negative effect of the TB prevalence and a positive effect of the TB incidence. Controlling for spatial effects of the TB measures leads to a decrease in the absolute value of the own raion effects, but informs us about a strong spillover of TB incidence and prevalence at a broader regional level, such as oblast. It demonstrates that the labour markets in Ukraine are mobile and defined at an oblast, rather than raion level. Moreover, any policy intervention should be performed at least at an oblast rather than raion level.

**Table 3:** Estimated own and spatial effects of TB incidence and TB prevalence on productivity

	Log regional wage		Residual log regional TFP	
	SLX	SAR	SLX	SAR
	(1)	(2)	(3)	(4)
Spatial lag of dependent variable		0.5326***		1.1652***
		(0.0442)		(0.1765)
Log of TB incidence	0.0397***	0.0143*	0.0401***	−0.0094
	(0.0086)	(0.0081)	(0.0124)	(0.0150)
Spatial lag (Log of TB incidence)	0.1221***	0.0613***	0.0838***	−0.0330
	(0.0127)	(0.0126)	(0.0182)	(0.0260)
Log of TB prevalence	−0.0216*	0.0135	−0.0757***	0.0564**
	(0.0122)	(0.0115)	(0.0175)	(0.0272)
Spatial lag (Log of TB prevalence)	−0.0491***	−0.0446***	−0.0608***	0.0446**
	(0.0108)	(0.0098)	(0.0154)	(0.0227)
Prison × year	−0.0184***	−0.0187***	−0.0192***	−0.0076*
	(0.0025)	(0.0022)	(0.0035)	(0.0041)
Prison× year in neighbouring raions	−0.0128***	−0.0041	−0.0246***	−0.0056
	(0.0043)	(0.0040)	(0.0062)	(0.0071)
Number of observations	3555	3555	3555	3555
Number of id	609	609	609	609

Notes: Spatial-econometric estimates—results come from raion-fixed-effects models that include (i) an spatial lag in X (SLX) specification with spatial lags of the TB variables, and (ii) a spatial autoregressive model (SAR) specification that additionally contains a spatial lag of the dependent variable. The weights matrix **W** is row-standardised first-order contiguity (raions that share a border). All right-hand side variables in all regressions are lagged 1 year; controls include education, mortality, unemployment, urbanisation, population density, prison, and—only for TFP—export/import shares. Although the IV point estimate of ρ exceeds unity, a Wald test fails to reject ρ=0.99 ($\chi ^2(1)=0.99$, ρ=0.321), indicating that ρ is not statistically distinguishable from a stable near-boundary process. We therefore interpret the SAR-lag estimate cautiously and rely on SLX/SEM specifications for spatial robustness. Robust standard errors in parentheses *p < 0.1; **p < 0.05; ***p < 0.01

The effects are statistically significant and economically important. A one percent increase in TB incidence in a raion and all adjacent raions is associated with a 0.1618% (0.0397+0.1221) higher average wages, while a one per cent **increase** in the TB prevalence in the same raions leads to 0.0707% (−0.0216−0.0491) **decline** in wage. For productivity, the effects are 0.1238% (0.0401+0.0613) and −0.1365% (−0.0757−0.0446) respectively.

Once we control for the spatial effects of wages in column (3), by estimating the SAR model, we observe that the wages are strongly spatially interdependent. The impacts of incidence and prevalence remain consistent with the previous results, consequently having significantly positive and significantly negative effects on the outcomes of interest. However, it would be misleading to compare the effect of the coefficients between these two models. Under the spatial autocorrelation model, the effect of a one-unit change in an explanatory variable *x* in raion *i* will have an impact on the dependent variable *y* not only locally, but also globally on all other regions and can be presented as a vector of outcomes.

14 $$\begin{aligned} \Delta y = (I-\rho \times W)^{-1}\times \beta _{x}\times \Delta x_i \end{aligned}$$

where *I* is an N×N the identity matrix, $\Delta x_i$ is a vector where the i-th unit is equal to one and the rest take values of 0, and Δy is the vector of outcomes. Given that the coefficient *rho* is estimated as 0.5326, a one per cent increase in the TB incidence level in all raions would lead to

15 $$\begin{aligned} \Delta ln(Regional Wage) = (1-0.53)^{-1} \times (0.0143+0.0613) = 0.1617 \end{aligned}$$

percent changes in regional wages in all regions. Likewise, a one per cent change in the TB prevalence level would change regional wages by

16 $$\begin{aligned} \Delta ln(Regional Wage) = (1-0.53)^{-1} \times (0.0135-0.0446) = -0.0665 \end{aligned}$$

Comparing these full impact elasticities of the SAR model in column (2) with the elasticities of the SLX model in column (1), we conclude that the two different specifications of the spatial models lead to quantitatively similar conclusions about the impact of the TB incident and prevalence on wages. However, the first model allows us to decompose the effect into a) direct local effect and b) spatial effect of the TB spillover from the neighbouring regions. We conclude that the impact of the TB variables on local wages is about 25–30% caused by the local situation in the raion, and the remaining effect is caused by the situation in the adjacent raions and oblast level.

For the TFP, the estimated spatial lag of Residual Regional TFP is greater than 1. We also estimated a model with inverse distance weights, as well as the Spatial Durbin model, where we included spatial lags of explanatory variables. In all cases, the estimated spatial coefficient was slightly above one, yet the test did not reject the hypothesis that the spatial process is stationary. Hence, on the one hand, we are unable to calculate the marginal effects that would be comparable with our baseline model, and it makes it impossible to comment on the sign and significance of the coefficients in the regression model because $(I-\lambda W)^{-1}$ does not have an economic interpretation. On the other hand, there is no evidence of a non-stationary spatial process. We therefore interpret the SAR-lag estimate cautiously and rely on SLX/SEM specifications for spatial robustness.

To conclude, while we explore the spatial lag models, our preferred specification remains the standard fixed effects model. This choice is grounded in both empirical and interpretive considerations. First, spatial models impose additional structure on the data, which can obscure the direct interpretation of coefficients, particularly when the focus is on estimating average regional effects attributable to TB burden.

Second, the spatial structure in our data reflects geographic contiguity rather than economic or social networks, which may not accurately capture the channels through which TB affects productivity. Finally, the inclusion of spatial lags does not substantially alter the magnitude or direction of our key estimates, suggesting that spatial dependence, while present, does not bias the core findings. For these reasons, we retain the fixed effects model as our preferred baseline for inference and policy simulation.

### Discussion

This section evaluates the robustness of our estimates and explores whether the observed productivity effects of TB vary across key firm characteristics (heterogeneity of the effects) and interprets the findings. These checks are important for assessing the internal validity and broader applicability of our findings. We take the fixed effects specification in Table 2 as a preferred baseline specification (row 1 “Main”) because it allows for a direct interpretation of the coefficient estimates and straightforward exploration of their heterogeneity.

Table 4 offers a number of sensitivity checks on the preferred specification for both the average regional wages (Columns 1–3) and the average regional TFP (Columns 4–6). Each row corresponds to a separate regression reporting only the estimates of the coefficients and standard errors for the incidence and prevalence of tuberculosis. We explore exclusion of controls for education, replacing linear time trend with the time dummies, including a specification where the indicator for prison is interacted with the set of time dummies, adding controls for population age structure, industry structure, firm characteristics (share of state ownership, average firm age, average firm size), as well as excluding raions within top 5% and bottom 5% of the population density distribution. As could be seen, in all cases there is no discernible difference in either the direction or the magnitude of the estimates. The only exception is in the specifications that replace the linear time trend with the set of time dummies, where in the wage regression, the coefficient for the TB prevalence becomes larger in magnitude, and the coefficient on TB incidence is somewhat attenuated. In the TFP regression, both coefficients are attenuated. Despite those differences, the stability of coefficients across most specifications supports the reliability of our main results and suggests that the estimated effects are not driven by omitted variable bias or specific modeling assumptions.

**Table 4:** Sensitivity analysis with regards to specification

	Log regional wage			Residual log regional TFP		
	Log of TB incidence	Log of TB prevalence	*N* obs	Log of TB incidence	Log of TB prevalence	*N* obs
	(1)	(2)	(3)	(4)	(5)	(6)
Main specification $$^{1}$$	0.0548^***^$$^{2}$$	-0.0333^**^	3555	0.0507^***^	-0.0895^***^	3555
	(0.0095)	(0.0148)		(0.0148)	(0.0187)	
Main – education	0.0545***	-0.0353**	3555	0.0494***	-0.0867***	3555
	(0.0096)	(0.0147)		(0.0151)	(0.0188)	
Main with year dummies	0.0352***	-0.0661***	3555	0.0354***	-0.0664***	3555
	(0.0082)	(0.0125)		(0.0082)	(0.0125)	
Main + prison × year dummies	0.0339***	-0.0654***	3555	0.0582***	-0.1402***	3555
	(0.0082)	(0.0124)		(0.0155)	(0.0192)	
Main + industry structure	0.0419**	-0.0451*	3555	0.0513***	-0.0896***	3555
	(0.0173)	(0.0256)		(0.0148)	(0.0188)	
Main + % working-age population	0.0498***	-0.0340**	3551	0.0455***	-0.0901***	3551
	(0.0096)	(0.0144)		(0.0148)	(0.0185)	
Main + firm characteristics	0.0540***	-0.0320**	3555	0.0517***	-0.0929***	3555
	(0.0095)	(0.0149)		(0.0148)	(0.0189)	
Main + firm chars + ind. structure	0.0542***	-0.0337**	3555	0.0523***	-0.0934***	3555
	(0.0092)	(0.0140)		(0.0148)	(0.0189)	
Main excl. top/bottom 5% pop. density	0.0545***	-0.0371**	3217	0.0501***	-0.1011***	3217
	(0.0100)	(0.0159)		(0.0160)	(0.0202)	
	Abortions	Live births	*N* obs	Abortions	Live births	*N* obs
“Placebo treatment”	0.0084	-0.0066	1762	-0.0116	-0.0190	1762
	(0.0081)	(0.0072)		(0.0177)	(0.0159)	

Notes: Right-hand side variables in all regressions are lagged 1 year; controls include education, mortality, unemployment, urbanisation, population density, prison and—only for TFP—export/import shares. Firm characteristics include regional averages of firm age, size, and state ownership share. Industry structure at raion level controls for share of labour in agriculture, industry and services. Each row re-estimates the baseline fixed-effects model from Table 2: the “Main” row already controls for education, mortality, unemployment, urbanisation, population density, and (for TFP only) export/import shares plus year dummies; the subsequent rows add or drop the covariates indicated in the stub. Robust standard errors in parentheses *p < 0.1; **p < 0.05; ***p < 0.01

As a placebo-like exercise, we examined whether other regional health outcomes predict wages through similar channels. Abortion rates reflect health system engagement and quality but do not generate sustained caregiving demands, yet they also do not represent any risks to those around the affected person, like in the case of TB incidence. Live birth rates represent health events requiring care, and potentially affecting productivity in similar ways as serious illnesses in the family. The last section of Table 4 shows that neither variable exhibits a statistically significant association with wages such as we observed for TB incidence and prevalence. This is consistent with our interpretation that it is the prolonged care burden of tuberculosis and its contagious nature rather than generic regional health variation that drives the productivity effects.

**Table 5:** Sensitivity analysis with different approaches to TFP measurement

	Residual regional TFP - FE					Lab. prod
	rtfp	op1	op1_acf	lp	lp_acf	LP
	(1)	(2)	(3)	(4)	(5)	
Log of TB incidence	0.0506***	0.0504***	0.0493***	0.0517***	0.0484***	0.0918***
	(0.0148)	(0.0140)	(0.0138)	(0.0145)	(0.0141)	(.0283)
Log of TB prevalence	−0.0892***	−0.0971***	−0.0986***	−0.0932***	−0.0886***	-.1005***
	(0.0187)	(0.0177)	(0.0174)	(0.0188)	(0.0178)	(.0422)

Notes: Right-hand side variables in all regressions are lagged 1 year; controls include education, mortality, unemployment, urbanisation, population density, prison, and export/import shares. Alternative TFP measures – All columns replicate the fixed-effects regression for residual regional TFP, but use five alternative firm-level productivity estimates: FE-residual (rtfp), Olley–Pakes (OP, 1996) (rtfp op1), OP with Ackerberg, Caves, and Frazer (ACF, 2015) correction (rtfp op1 acf), Levinsohn–Petrin (LP, 2003) (rtfp lp), and LP with ACF correction (rtfp lp acf), and labor productivity (LP). Regional TFP is always the labour-weighted average of firm TFP. Controls, sample, and significance coding are identical to Table 2. Robust standard errors in parentheses *p < 0.1; **p < 0.05; ***p < 0.01

**Table 6:** Heterogeneity analysis by firm characteristics for regional wages

	Log of average regional wage - FE			
	Firm size	Share with >50% state ownership	Share of employment in agriculture	Share of employment in services
	(1)	(2)	(3)	(4)
Log of TB incidence	0.0644	0.1675***	0.2633***	0.2633***
	(0.0928)	(0.0186)	(0.0548)	(0.0548)
Log of TB prevalence	−0.3286***	−0.3602***	−0.3231***	−0.3231***
	(0.1150)	(0.0282)	(0.0722)	(0.0722)
Explored dimension	−0.0544	−7.9899**	−0.0344	−0.1666
	(0.1153)	(3.3623)	(0.4944)	(0.6217)
× Log of TB incidence	0.0207	0.2213	−0.0638	−0.3175***
	(0.0173)	(0.4608)	(0.0667)	(0.1059)
× Log of TB prevalence	−0.0061	1.1149**	−0.1541	0.3082**
	(0.0194)	(0.5217)	(0.0953)	(0.1225)

Notes: The fixed-effects specification from Table 2 (Column 2) is augmented by the interaction terms with the variables that reflect average firm characteristics in the region. “Explored dimension” refers to the moderating variable indicated in each column header. Robust standard errors in parentheses. *p < 0.1; **p < 0.05; ***p < 0.01

**Table 7:** Heterogeneity analysis by firm characteristics for total factor productivity

	Residual regional TFP - FE			
	Firm size	Share with >50% state ownership	Share of employment in agriculture	Share of employment in services
	(1)	(2)	(3)	(4)
Log of TB incidence	0.2160**	0.1194***	0.1310*	0.1310*
	(0.0881)	(0.0173)	(0.0723)	(0.0723)
Log of TB prevalence	−0.6739***	−0.2895***	−0.0467	−0.0467
	(0.1105)	(0.0246)	(0.0905)	(0.0905)
Explored dimension	−0.3082***	−10.3952***	1.7955***	1.2112
	(0.1133)	(2.9370)	(0.5304)	(0.8417)
× Log of TB incidence	−0.0178	−0.1609	0.1110	−0.3258*
	(0.0174)	(0.4851)	(0.0789)	(0.1555)
× Log of TB prevalence	0.0719***	1.9142***	−0.4894***	0.0625
	(0.0190)	(0.5116)	(0.1045)	(0.1827)

Notes: The fixed-effects specification from Table 2 (Column 4) is augmented by the interaction terms with the variables that reflect average firm characteristics in the region. “Explored dimension” refers to the moderating variable indicated in each column header. Robust standard errors in parentheses. *p < 0.1; **p < 0.05; ***p < 0.01

Given that there exist a number of approaches to the estimation of the total factor productivity, we also re-estimated the preferred specification using the measures from these different approaches. Table 5 presents the results with the TFP estimated by the fixed effect (column 1), Olley and Pakes (Olley and Pakes 1996) in column (2), ACF (Ackerberg et al. 2015) in columns (3) and (5), and Levinsohn and Petrin (Levinsohn and Petrin 2003) in column (4). It also reports the estimates of labor productivity as an alternative measure of firm productivity (Cardullo et al. 2024)^5^ in column (6). As can be seen, the estimates show no detectable differences between the TFP measures of the five approaches used in the productivity literature, or the simpler labor productivity measure, which shows robustness of our results to the different measures of productivity used in the literature.

**Fig. 6:**
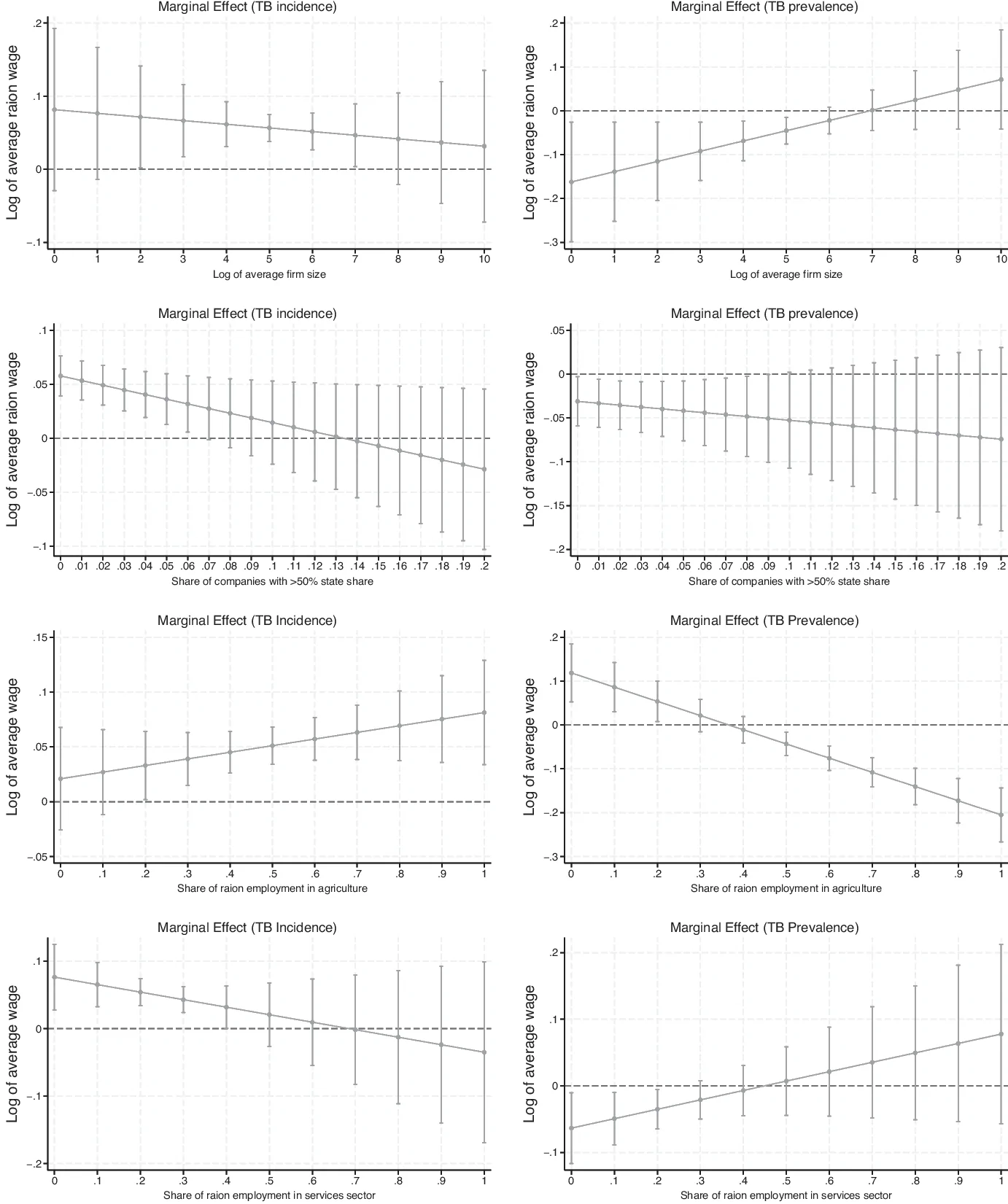
Heterogeneous effects of TB on regional wages

**Fig. 7:**
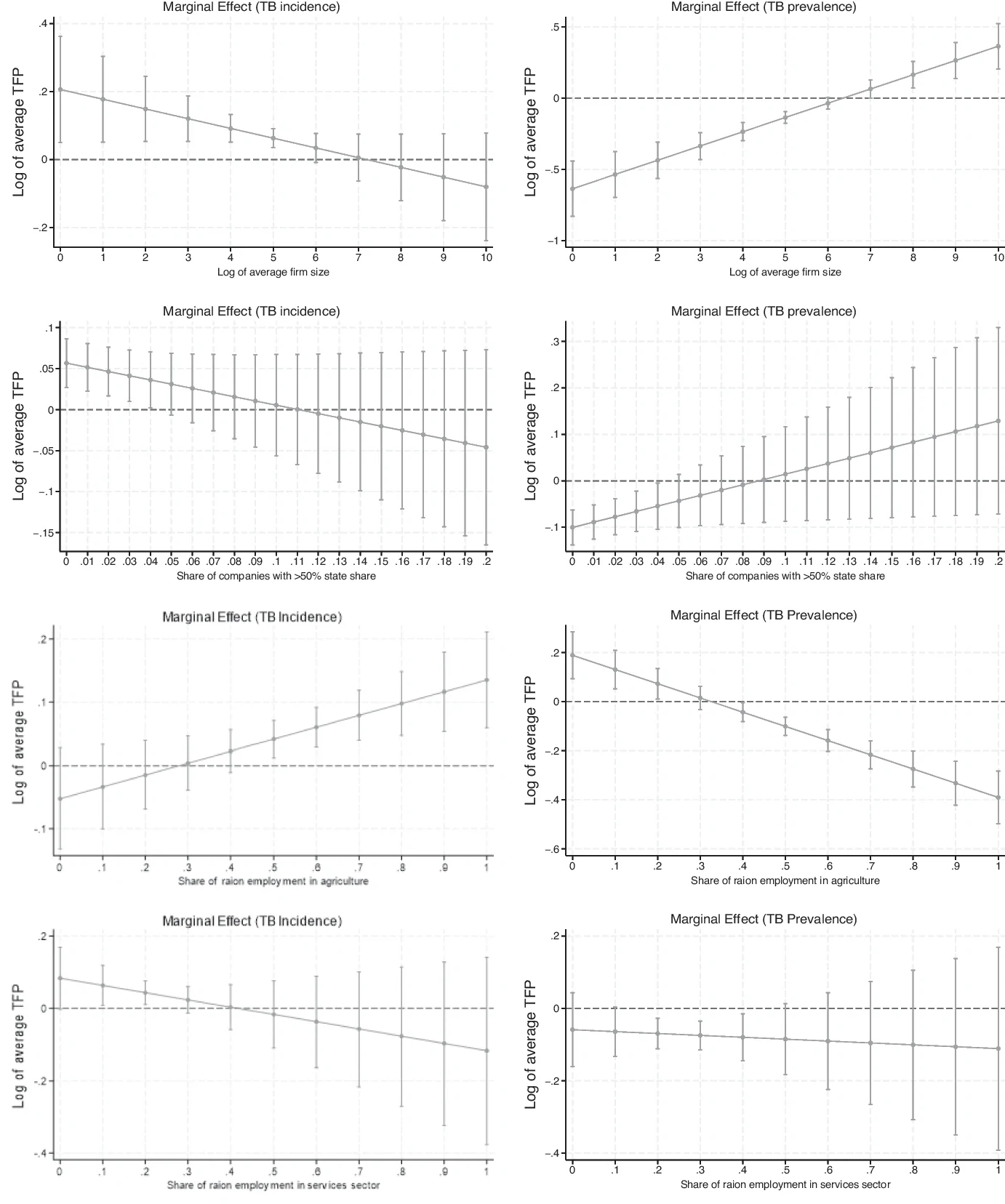
Heterogeneous effects of TB on total factor productivity

To better understand the factors which may interact with the TB effects, we explored the heterogeneity of the effects of TB along several dimensions: depending on the average firm size, state ownership share, and industrial structure. Tables 6 and 7 present the results of those estimations. While the first two columns represent separate regressions interacting the set of TB variables with the average firm size in a raion and with the share of firms with the state ownership of equal or greater than 50%, the last two columns refer to the same regression where the interaction is with the raions’ industrial structure. The interpretation of the coefficients and their statistical significance is convoluted by the fact that the interactions are with continuous variables. For example, they may tell us whether the effect is larger or smaller in more agricultural regions, but not how much larger, nor at what threshold the effect becomes statistically distinguishable from zero. Marginal effects plots presented in Figs. 6 and 7 convey this information directly and intuitively.

We find that the effects of TB prevalence and incidence are largest in absolute value within raions with the smaller average firm size, smaller state ownership share, and larger share of firms in agriculture. This is consistent with the theoretical predictions, as the small size of the firms and limited state ownership are the prerequisites of competitive markets. With regard to the share of agriculture, the finding of the largest effects in raions which are predominantly agricultural is also consistent with the theory, as this is the sector which is most homogeneous in terms of workforce and technology, as well as having the settings with the most transparency in terms of workers’ effort (e.g., in piece-rate contracts) as compared to services or industry sectors. From a policy perspective, this suggests that TB mitigation strategies may be particularly beneficial for more competitive business environments—smaller private enterprises in the agricultural sector.

The robust finding of opposite signs on incidence and prevalence across specifications, outcome measures, and subsamples provides empirical support for the theoretical distinction between risk compensation and productivity loss channels.

**Table 8:** Comparison of current expenditures on TB-related programs from all sources and individual productivity losses associated with a 10% increase in TB prevalence

	2014	2009
Gross domestic product (UAH)	1,586,900,000,000	947,042,000,000
Employment (persons)	18,073,300	20,191,500
Average monthly wage	3368	1906
Considered decrease in TB prevalence (%)	−10	−10
TFP elasticity with respect to TB prevalence	−0.0893	−0.0893
Wage elasticity with respect to TB prevalence	−0.0333	−0.0333
Total national wage bill	730,450,492,800	461,819,988,000
Gain from fighting TB in terms of wages (UAH)	2,432,400,141	1,537,860,560
Gain from fighting TB in terms of wages (% of GDP)	0.15	0.16
Gain from fighting TB in terms of TFP (% of GDP)	0.89	0.89
Total gain from fighting TB (% of GDP)	1.05	1.06
TB-related expenditure (UAH)	568,836,440	n/a
TB-related expenditure (% of GDP)	0.04	

Cost-benefit summary – National GDP, employment, and average wage figures are taken from State Statistics Service aggregates for 2009 and 2014; monetary values are in current Ukrainian hryvnia (UAH). Gains are computed by applying the wage and TFP elasticities from Table 2 (−0.0333 and −0.0893) to a hypothetical 10 % drop in TB prevalence, with programme spending combining domestic and donor funds. Percentages are expressed as shares of GDP; “total gain” is the sum of wage and TFP effects

Table 8 provides an example of the calculation of the overall country-wide benefits of containing the TB epidemic in terms of worker and firm productivity. This comparison highlights that even modest improvements in TB control could deliver gains that exceed current TB-related spending by an order of magnitude. These calculations are provided using the country-level data on GDP, the number of employed individuals, and the average monthly wage rate. Applying the estimated wage and TFP elasticities with respect to the TB prevalence to the 2014^6^ figures shows that, ceteris paribus, a 10% reduction in TB prevalence would result into a 1.05% increase in GDP (0.89% increase via an increase in TFP and 0.15% via an increase in the individual productivity as measured by the average monthly wages). These estimates are somewhat smaller—0.97% (0.66% increase via an increase in TFP and 0.31% via an increase in the individual productivity) based on the regression with time dummies. The numbers are similar for the year 2009. Note that the ceteris paribus condition means keeping constant the incidence rates of tuberculosis and the death rates at the raion level. This implies that the reduction in TB prevalence can only come from the improvement in treatment and successful recovery of patients. To put things into perspective, the total TB-related expenditure in the country (jointly funded by the state and international donors, including contributions from the Global Fund) accounted for only 0.04% of GDP that year. This points to a significant underinvestment relative to the scale of returns achievable, even before accounting for the much larger share of costs related to premature mortality.

The only other major infectious disease that has severely affected Ukraine is HIV/AIDS. However, no comparable productivity-based estimates exist in the economic literature for Ukraine. In the context of Sub-Saharan Africa, Waziri et al. (2015) estimate that a 10% reduction in HIV prevalence leads to a 0.14% increase in GDP—substantially smaller than the 1.05% increase associated with a 10% reduction in TB prevalence estimated in this paper. Similarly, Gallup and Sachs (2001) show that a 10% reduction in malaria (as measured by a malaria index) is associated with a 0.3% increase in economic growth. While comparisons across these studies are necessarily limited due to differences in country samples, health indicators, and methodological approaches, the relatively large estimated return to TB control highlights the long-term underinvestment in TB, especially in contrast to the more substantial global funding directed at HIV/AIDS and malaria in recent decades (Bloom et al. 2022).

The interpretation of the economic effects from changes in the TB incidence rates is a much more complicated exercise. First of all, a reduction of the documented incidence rate may not imply a reduction in the risk levels, as it may indicate the failures of the diagnostics and the contact tracing systems. Likewise, an increase in the documented TB incidence rate may actually be a sign of an improvement in the TB diagnostics. Second, as mentioned above, the economic effects of the reduction in the TB incidence would play out via a reduction in costs of production and thus making regional products more competitive at the national and international levels. Capturing these effects would require a sophisticated modeling of the markets for various industries at regional, national, and international levels, which is beyond the scope of this paper.

Our findings have several important policy implications extending beyond the case of Ukraine. First, we show that TB prevalence and incidence capture conceptually and empirically distinct aspects of disease burden: prevalence reflects the chronic health and economic drain from existing cases, while incidence captures the risk of new infections. Given the correlation between the two measures and theoretically opposing effects on productivity, which we have confirmed empirically, failing to account for both indicators can lead to biased estimates and misinformed policy priorities. This highlights the critical need for national and subnational surveillance systems to collect, report, and analyze both measures routinely.

Second, the study documents sizable indirect productivity losses associated with underscoring that the economic burden of tuberculosis extends far beyond direct medical costs and premature mortality. These findings advocate for a wider perspective in cost-effectiveness analyses for informed policy decision-making, recognising the hidden productivity penalties of an ongoing TB epidemic.

Third, even when focusing solely on indirect productivity effects, our findings reveal substantial underinvestment in TB control. While the specific magnitudes may differ across countries, our estimates clearly call for a reassessment of the full economic costs of the TB epidemic, even in settings where TB mortality is low. In today’s world of increasing cross-border displacement driven by armed conflict and climate-related disasters, even countries with strong healthcare systems are not immune to the productivity losses associated with TB risks. These indirect economic consequences, often overlooked in traditional cost-benefit assessments, should be considered in shaping both national and international strategies for disease control and health system preparedness.

Finally, the spatial patterns we document point to broader regional effects caused by uncontained epidemics, which can affect neighboring jurisdictions. This calls for coordinated subnational policy responses, especially in decentralised systems, and justifies targeted investment in high-burden areas to prevent contagion of both disease and economic disadvantage.

## Conclusions

This study is the first to quantify the indirect productivity costs of tuberculosis by linking epidemiological dynamics to both worker and firm-level productivity outcomes. Using a uniquely rich administrative dataset from Ukraine covering 609 raions between 2003 and 2009, we combine regional TB prevalence and incidence data with the detailed socio-economic statistics to estimate the economic burden of the epidemic beyond direct treatment costs.

Our findings show that TB prevalence negatively affects both worker productivity measured as an average regional wage and the total factor productivity (TFP), which supports the health-productivity link explored by Becker, Grossman, and Strauss and Thomas. In contrast, TB incidence appears to command a wage premium, consistent with the Rosen’s theory of compensating wage differentials. This points to a structural inefficiency, where higher labour costs in high-risk areas may hinder firm competitiveness. The TB-related TFP premium requires further empirical investigation and theoretical consideration. Potentially, the nature of this premium is similar to that of compensating wage differentials with regard to managerial talent, entrepreneurship, and creativity. Importantly, both productivity and compensatory effects are more pronounced in regions which exhibit characteristics of more competitive environments (smaller firms, lower share of state ownership) and in more labour-intensive sectors.

Our spatial analysis further reveals significant cross-regional spillovers, highlighting the contagious nature of TB and the need for coordinated containment strategies beyond local administrative boundaries.

The fact that a 10% decrease in TB prevalence could lead to a 1.05% increase in GDP (0.33% through higher individual productivity (wages) and 0.89% through higher total factor productivity) highlights the stark contrast with the TB-related spending of only 0.04% of GDP in 2014. This is a clear evidence of substantial underinvestment in TB control, even when considering only the indirect productivity effects.

The implications of this study extend well beyond Ukraine. For post-Soviet countries, the methodology provides a replicable, ready-to-use framework for estimating the economic burden of TB and supporting evidence-based advocacy. For policymakers in both low- and high-income countries, our findings highlight the need to consider indirect productivity losses in evaluating the full economic cost of TB, especially amid growing global mobility and displacement due to climate-related disasters and armed conflicts, and the need to routinely collect data both TB prevalence and TB incidence. Finally, for researchers, this study offers a basis for expanding the literature on the economic consequences of infectious diseases by integrating high-resolution subnational data, spatial analysis, and highlighting the importance of taking into account different dimensions of the parameters describing the epidemic.

## Data Availability

Data used in this analysis combined both administrative health and socio-economic statistics for administrative units, as well as TFP estimates from the firm-level data. The analytic data set and the Stata code are available from the corresponding author upon request.

## Footnotes

1 Standard treatment takes at least 6 months and often extends to a year (Connolly et al. 2007)

2 http://data.worldbank.org/indicator/SH.TBS.INCD?locations=UA

3 https://www.ioc.gov.ua/analytics/dashboard-vpo.

4 https://reporting.unhcr.org/operational/situations/ukraine-situation.

5 This alternative measure is calculated as total firm sales net of the cost of materials divided by the number of employees and aggregated to the regional level (raion) using employment-weighted averages, similar to our TFP aggregation.

6 First year when the total estimate of the TB expenditure was made available.
